# Sharing the Same Reality, Healthy Relations Between Colleagues at Work: A Meta-Synthesis

**DOI:** 10.1177/23779608231207239

**Published:** 2023-10-16

**Authors:** Erebouni Arakelian, Gudrun Rudolfsson

**Affiliations:** 1 560570 Faculty of Health and Occupational Studies, Department of Caring Sciences, University of Gävle, Gävle, Sweden; 2 174469 Department of Surgical Sciences, Uppsala University, Uppsala, Sweden; 3 Faculty of Nursing and Health Sciences, Nord University, Bodø, Norway; 4 Division of Nursing, Department of Health Sciences, University West, Trollhättan, Sweden

**Keywords:** health, home, humanistic perspectives, interprofessional, meta-synthesis, relationships, well-being, work environment

## Abstract

**Introduction:**

Good relations are important at work, leading to well-being among coworkers. Since the latest research in nursing is mostly about bullying, and lateral violence, it was important to study what healthy relations mean.

**Objectives:**

The aim was to identify and synthesize qualitative studies that describe healthy relations, creating health and well-being between colleagues at work.

**Methods:**

A meta-synthesis approach, inspired by Sherwood's steps of analysis, was chosen for this study. Ten articles from three continents, comprising 230 participants, were included.

**Results:**

Two themes were identified as follows: (a) creating a mutual bond on a personal level and a permissive atmosphere through the human warmth; and (b) sharing togetherness in a greater whole, offering unconditional help and devoting themselves to taking care of each other. An overarching metaphor implicating the home/homeness was abstracted from the two themes: “Healthy relations between colleagues at work constitute the community as a common world, containing feelings of being at home, implying acting in an expression of the ethos and dignity, a culture created that makes the ideal form of healthy relations visible.”

**Conclusion:**

Nurses find their meaning when they work in a permissive environment, and when they are allowed to be themselves. Such meaning can be found through their engagement with one another, by offering each other unconditional help. Good relationships lead to a place they call their home, where there is trust and friendship.

## Introduction

Good relations are important at work, leading to well-being among coworkers. Since the latest research in nursing is mostly about bullying, and lateral violence, it was important to study what healthy relations mean.

## Review of Literature

In her collected works, Eriksson (2018) presents specific premises based on the concept of health, namely salubrity, liveliness, and well-being that constitute the substance of health, that is, a person's capacity and internal support. An integrated state of salubrity, liveliness, and well-being presupposes awareness, that is, experience of a sense of significance, as well as the ability for autonomy versus dependence. Well-being describes the person's perceived health and can thus be considered as denoting the person's degree of awareness, experience of goals, and sense of significance. Eriksson (2018) states that the context of health constitutes the human being as a whole and encompasses the room dimension, which changes over time. This statement reflects that health is relative in relation to time and space (Eriksson, 2018).

Svenaeus (2019) views health as a phenomenon of homelikeness, with being-in-the-world. Good health is essentially concealed from us, but despite its hidden character, health shows itself in a general feeling of well-being, where such a feeling means we are open to being together with one's fellow human beings, of active engagement in one's everyday tasks (Svenaeus, 1999). Being together with one's fellow human beings implies health and that a common world emerges (Lindberg & Rudolfsson, 2019). What is said, together with one's fellow human beings, is a sign of feeling at home, in a communion, where a dialogue is created, full of life and meaning. A common world is a world where harmony, health, and the idea of awe for human dignity carry the day to attain the feeling of true homecoming. According to Lindberg and Rudolfsson (2019), the common world creates a movement inside each human being, where the boundless life-giving time represents the settlement where the movement of time is inhabited, like coming home.

A person's health and well-being are essential to professional growth and require curiosity, humility, self-awareness, and a motivation to master (Ramani et al., 2019). Previous research highlights that communication is adapted to enhance the dialogue between nurses, affecting the ability to understand each other (Hayward, 2021). Emotional intelligence is central to effective teamwork (McCallin & Bamford, 2007), in the view of self-management, social awareness, and social skills. According to Milliken and Grace (2017), nurses who are ethically sensitive are likely to be more professionally responsible, in terms of recognition of the ethical content of an action. Moreover, James-Scotter et al. (2019), in an operating room setting, identified that recognition and positive communication improve job satisfaction, confidence, and performance (James-Scotter et al., 2019). Such an environment begins with caring for one another, based on a philosophy of fostering trust and empowering and respecting one another (France et al., 2011). According to Wärnå et al. (2007) health is understood as a struggle for finding one's own value as a human being. Wärnå et al. (2007) maintain that work and happiness belong together in the same way as pride; thus, keeping promises and life-lust form the meaningful content of health. In addition, by treating human beings in a dignified way within occupational health care and being honest, pride will be fostered and hopefully reshaped of those who have lost it in working life.

Although there is some research and an extensive theorizing about healthy relationships, previous results are predominantly about bullying and poorly functioning relationships in working life (Bigony et al., 2009; Lögde et al., 2018; Oh et al., 2016) The frequency of witnessing coworker bullying weekly varies in different studies (Chipps et al., 2013; Villafranca et al., 2017). Lögde et al. (2018) described that act of incivility or being “systematically frozen out” among operating room nurses led to quitting one's job. Furthermore, acts of ignoring nurse colleagues or verbal or psychological violence were also described (Chipps et al., 2013; Lang et al., 2022).

These results were confirmed also by Oh et al. (2016) and Bigony et al. (2009) who meant that incivility or bullying towards nurse colleagues affected patient outcome negatively.

### Purpose

The purpose was therefore to identify and synthesize qualitative studies that describe healthy relations, creating health and well-being between colleagues at work.

## Methods

### Design

Meta-synthesis is a methodology for synthesizing existing qualitative research findings by focusing on different aspects of the chosen phenomenon; furthermore, it seeks to expand the possible interpretations of findings, and it creates a transformed whole as well as a new understanding (Bondas & Hall, 2007; Sherwood, 1999). In text interpretation, the text is seen as a whole in which the dialogue between the reader and the text is intended to contribute towards a new understanding through a dialectic movement between the whole and the parts (Gadamer, 1975). The goals of meta-synthesis are theory building, theory explication, and theoretical development. According to Sherwood (1999), the starting point is two crucial steps as follows: identifying the domain of study and establishing the inclusion criteria for selecting the articles for the study. Furthermore, the research question must be broad enough to be interesting, but small enough to manage.

### Sample: Search Strategy, Article Selection, and Quality Appraisal, Inclusion and Exclusion

A comprehensive search of the electronic databases PUBMED and CINAHL, SCOPUS and ACADEMI SEARCH ELITE was conducted during Autumn 2021. The search was limited to studies published between January 1, 2012 and December 31, 2021. The following key terms were combined with operators: “health promoting” or “health promoting relations” or “relations” or “nursing” or “perioperative” or “emergency” or “intensive care” and (“qualitative study” or “qualitative research”). The following additional keyword searches were also used: “Interpersonal relations/relationships,” “encounter between colleagues,” “respect,” “dignity,” “cooperation between colleagues,” “appreciation,” “friendship at work,” and “fellowship.” To ensure accuracy, a second-round search was carried out in January/February 2022. Inclusion criteria were articles concerning good relations between nurse colleagues; exclusion criteria were articles published earlier than 2012 including bad relations (described as nurse-to-nurse bullying and incivility), relations between patient and nurses, doctors/physicians and nurses, and managers and nurses as well as literature about COVID-19.

**Figure 1.**
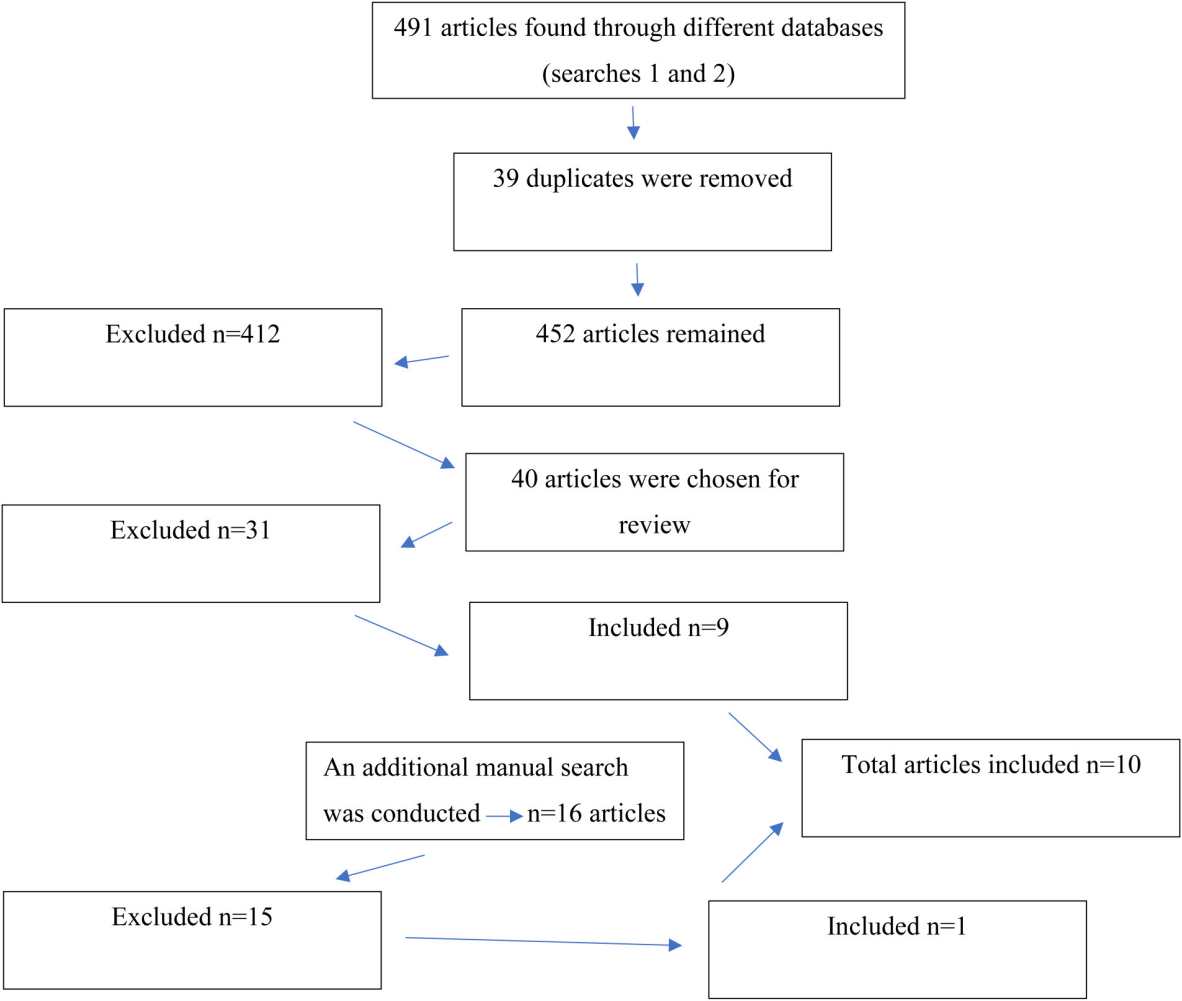
Search strategy.

Figure 1 outlines the study selection process. The initial electronic database search retrieved a total of 491 articles. All the search records were compared, and duplicates were removed. After removing duplicates, 452 articles remained. Next, the title and abstract screens of 452, 412 articles were eliminated. The full text of the remaining 40 studies was then assessed for eligibility. Among these, 31 were excluded because they were not pertinent to the aim (*n *= 15) or did not meet the inclusion criteria (*n *= 16). Nine articles were included in the analysis from the first search. In addition, a manual search was conducted, leading to 16 new articles, of which all were read through. Thereafter, 15 were excluded (due to not meeting the inclusion criteria), and one was included.

The first and the second authors performed the search independently, and both checked the study's selection process for accuracy. Critical Appraisal Skills Program Qualitative checklist (CASP) was used to assess the methodological quality of the included studies (*CASP Checklists - Critical Appraisal Skills Programme*, n.d.). CASP consists of 10 questions with “yes,” “no,” or “can’t tell” responses (Table 1). They discussed their CASP evaluations together, and a consensus was reached.

**Table 1.** Critical Appraisal of the Included Articles (CASP).

Article/CASP question	1	2	3	4	5	6	7	8	9	10
1	Y	Y	N	Y	Y	N	Y	Y	Y	Y
2	Y	Y	N	C	C	N	Y	N	Y	Y
3	Y	Y	N	C	Y	N	Y	N	Y	Y
4	Y	Y	N	Y	Y	Y	Y	Y	Y	Y
5	Y	Y	N	Y	N	Y	Y	Y	Y	Y
6	Y	Y	N	Y	Y	N	Y	Y	Y	Y
7	Y	Y	N	Y	Y	N	Y	Y	Y	Y
8	Y	Y	Y	Y	Y	N	Y	N	N	Y
9	Y	Y	N	N	Y	N	Y	Y	Y	Y
10	Y	Y	Y	Y	Y	N	Y	Y	Y	Y

Questions in CASP: 1. Was there a clear statement of the aims of the research? 2. Is a qualitative methodology appropriate? 3. Was the research design appropriate to address the aims of the research? 4. Was the recruitment strategy appropriate to the aims of the research? 5. Was the data collected in a way that addressed the research issue? 6. Has the relationship between researcher and participants been adequately considered? 7. Have ethical issues been taken into consideration? 8. Was the data analysis sufficiently rigorous? 9. Is there a clear statement of findings? and 10. How valuable is the research?. Y = yes; N = no; C = can’t tell.

The 10 included articles that were from 2017–2021 represent Asia (Iran), Europe (Norway, Denmark, and Sweden), South America (Brazil), Australia (Australia and Indonesia), and North America (Canada) (Table 2).

**Table 2.** The Included Articles in the Study, Their Aim, Methods, Participants, and Results.

	Manuscript title	Authors	Year of publication	Country	Participants	Method	Results
1	Perspective and experiences of operating room personnel	Bakhtiari S, Rakhshan M, Shahriari M, Sharif F.	2020	Iran	14 OR nurses and technicians, 2 surgeons, 2 anesthesiologists, 4 educators, 4 managers. The participants were between 26 and 51 years of age and they had 4–26 years of work experience.	Qualitative content analysis. Semi-structured individual interviews.	Four themes were found as follows: (a) enhance professional commintment; (b) strive for excellence; (c) respect for human dignity; and (d) enhance safety.
2	Communication and relationship dynamics in surgical teams in the operating room: An ethnographic study	Tørring B, Hoffer Gittell J, Laursen M, Steen Rasmussen B, Elgaard Sørensen E.	2019	Denmark	Participant observations of 39 surgical teams and 15 semi-structured interviews during a 10-month period in 2014 in two orthopedic operating units in a university hospital in Denmark.	Participant observations and semi-structured individual interviews. A deductively directed content analysis was carried out based on the theory of relational coordination.	Four types of collaboration representing different communication and relationship were identified as follows: (a) proactive and intuitive communication; (b) silent and ordinary communication; (c) inattentive and ambiguous communication; and (d) contradictory and highly dynamic communication.
3	Interpersonal relationships in the surgical unit from the perspective of nursing workers: An exploratory study	Trajano MFC, Gontijo DT, da Silva MW, De Aquino JM, Monteiro E.	2017	Brazil	Four of 40 nursing workers participated in the pretest of the data collection instrument and 25 nursing workers from both day and night shifts participated in the data collection. The participants were between 32 and62 years of age and, they had between 3 and 35 years’ experience in their profession.	Thematic content analysis.	Four were identified as follows: (a) proactive and intuitive communication; (b) silent and ordinary communication; (c) inattentive and ambiguous communication; and (d) contradictory and highly dynamic communication. The findings suggest a connection between communication and relationship dynamics in surgical teams and the level of complexity of the surgical procedures performed.
4	I stay—Swedish specialist nurses in the perioperative context and their reasons to stay at their workplace	Arakelian E, Rudolfsson G, Rask-Andersen A, Runeson-Broberg R, Wålinder R.	2019	Sweden	15 nurse (two men and 13 women aged between 43 and 63) specialists from four hospitals in Sweden.	Semi-structured individual interviews. Systematic text condensation, STC.	Three themes were identified as follows: (a) organizational stability contributed to low staff turnover, with good spirits between colleagues, representing everyone's equal value and resulting in a feeling of homelikeness; (b) sustained development in one's own profession; and (c) a humane head nurse who was at hand, who was a facilitator, who knew staff members, and eliminated obstacles for them.
5	Resources for work-related well-being: A qualitative study about healthcare employees’ experiences of relationships at work	Schon Persson S, Nilsson Lindstrom P, Pettersson P, Nilsson M, Blomqvist K.	2018	Sweden	23 healthcare employees (21 women, two men) in municipal health care. 18 AN, two RN, two OT, and one PT—had between 5 and 36 years’ experience in their current profession.	Qualitative individual interview study. Thematic analysis was used to analyze the data.	Two themes were found as follows: (a) being personal—a close interpersonal relationship to a care recipient—and (b) colleague belongingness—a sense of togetherness within the working group. Spending quality time together, providing long-term care and providing additional care were prerequisites for a close interpersonal relationship with care recipients. Trust, mutual responsibility and cooperation were prerequisites for a sense of togetherness within the working group.
6	Nontechnical skills in operating room nursing: Ethical aspects	Hanssen I, Smith Jacobsen IL, Skråmm SH.	2020	Norway	11 experienced perioperative/operating room nurses working in an operating unit at a Norwegian university hospital.	Qualitative individual in-depth interviews were conducted. The interviews were analyzed using Braun and Clarke's thematic analysis.	Three themes were found, namely as follows: (a) respect and care for the patient; (b) making the patient feel safe; and (c) respect within the perioperative team. Collaboration and communication within the team are closely connected to patient safety.
7	The development of nurses’ foundational values	Sastrawan S, Weller-Newton J, Brand G, Malik G.	2021	Indonesia/Australia	Individual interviews with 24 and focus group interviews with 23 participants were conducted.	Grounded theory.	Foundational values were achieved by family upbringing, professional nurse education, and organizational/institutional values reinforcement. These values are framed through religious lens, humanity perspective, and professionalism. This frames a unique combination of personal–professional values that comprise nurses’ values system. Values are transferred to other nurses either in a formal or informal way as part of one's professional responsibility and social interaction via telling and sharing in person.
8	The value of friendship in interprofessional healthcare team: A secondary analysis	Sedig K, Sibbald SL.	2021	Canada	Seven family health teams, 4 focus groups including 21 participants in total comprising respiratory therapists, clinical lead physicians, nurse practitioners, and providers in executive or administrative roles.	Secondary thematic analysis was conducted.	Participants valued their relationships with each other, and that they rely on one another. Empathetic listening, frequency of interaction, and emotional expression were mentioned regularly by participants. Three themes were identified as follows: (a) the ease with which participants could reach out to team members; (b) genuine enjoyment at the prospect of spending time with their team; and (c) the perceived normalcy of team members’ closeness.
9	Qualitative evaluation of regular morning meetings aimed at improving interdisciplinary communication and patient outcomes	Aston J, Shi E, Bullôt H, Galway R, Crisp J.	2005	Australia	Medical staff (*n* = 10), and nursing participants (*n* = 9)	Qualitative analysis	Four themes were identified as follows: (a) predictability: a nice way to start a day; (b) knowledge and perspectives: learning from each other; (c) relationsshipa and support: getting to know you; and (d) desired outcomes: makig a difference to staff, children, and families.
10	Salutary factors and hospital work environments: A qualitative descriptive study of nurses in Sweden	Nunstedt H, Eriksson M, Obei A, Hillström L, Truong A, Pennbrant S.	2020	Sweden	12 nurses in emergency medicine, specialist medicine, surgical care, and adult psychiatric care.	Qualitative content analysis.	Three themes, that is, (a) comprehensibility, (b) manageability, and (c) meaningfulness, and 10 subthemes were categorized as follows: job satisfaction and fun at work, acknowledgement and productivity, togetherness and team security, manageable workload, variable work and challenging situations, workplace and personal space balance, collaboration and supportive leadership, valued role and good work, commitment and involvement, and pride in the professional role.

### Qualitative Appraisal of the Included Studies

Most questions were raised in a sub-question to CASP 3 “to ensure quality assessment of the studies (sub-question: if the researcher has justified the design).” Such information was lacking in almost all of the included studies. In two studies, information about “Has the relationship between the researcher and the participants been adequately considered?” was lacking, and in the other two, the data analysis was not “sufficiently rigorous” (CASP 8 question).

### Phases in the Synthesis

The synthesis was performed in three phases, as described by Sherwood (1999). The first phase aimed to identify and extract key terms, concepts and findings that were especially descriptive of the healthy relations and well-being at work. The second phase strived to find a common language within which the findings could be translated into each other. Finally, the findings were synthesized into a new whole, in order to move beyond the near-sightedness of individual horizons (Vilhauer, 2010); in this case, the authors’ own understanding. The text interpretation was conducted by means of a constant dialogue between the two authors. Such a living dialogue allows for not only a fusion between the past and the present, that is, between the interpreters and texts, but also a fusion between two people living in the present. The authors’ historical horizons are based on a caring science perspective, characterized by caritas, human dignity, and caring relationships, that is, a caritative ethic (Eriksson, 1992).

In this study, the text to be synthesized came from 10 articles and contained data from 230 participants and 39 observations of 85 team members in surgical teams, aged between 26 and 62 years of age. One study lacked information about the participants’ age (Tørring et al., 2019). Four studies employed thematic analysis, four had used qualitative content analysis, one had a grounded theory approach, and two had a systematic text condensation as the analysis method. Data were collected by means of individual interviews, focus group interviews, and observations.

*The first phase* involved an interpretation of the existing themes, categories, and abstracted text that wove together the individual studies. The aim was to discover key terms, concepts and findings (Sherwood, 1999) referring to healthy relations and well-being at work, as well as to generate possible new interpretations in a dialectical manner. These key terms, concepts and findings were then carefully examined in the light of the authors’ pre-understanding in order not to draw any hasty conclusions and thus obstruct further understanding.

*In the second phase*, the various meanings were then organized within a meaning context, which involved comparing and contrasting, translating them into each other, by extracting them from their original context as a means to highlight and elevate them to the level of a common language (Sherwood, 1999). The search for meaning was constantly governed by three main questions as follows: What does the text say, what does it mean, and what is the deeper meaning imparted in the text?

*In the third phase*, common features emerged and themes were formulated, through new comparisons (Sherwood, 1999), which captured meanings and meaningful patterns of the prerequisites of healthy relations and well-being at work. These themes were further refined in an attempt to understand and reach an agreement on a new view as well as a more comprehensive, yet deeper whole for the purpose of elevating them to the level of a synthesized abstraction.

## Institutional Review Board Approval

To conduct a meta-synthesis does not require any ethical approval. However, ethical issues had been taken into consideration in the included studies.

## Results

### Sample Characteristics

Ten articles were included. These were published between 2017 and 2021, and represented Asia (Iran), Europe (Norway, Denmark, and Sweden), South America (Brazil), Australia (Australia and Indonesia), and North America (Canada).

### Research Question Results

Through the translation processes of the included studies, two main themes emerged, based on 37 subthemes concerning health and well-being between colleagues at work (Table 3). A final synthesis was presented as a metaphorical construction.

**Table 3.** The Subthemes and Themes Included in the Study.

Theme	Subthemes	In article(s)
Creating a mutual bond on a personal level and a permissive atmosphere through the human warmth	1- Understanding of each other	5, 9
	2- To open-up to one's colleagues	4, 9
	3- To have a friend at work	4
	4- Honest and friendly atmosphere	5, 6
	5- One dared to show one's inner feelings	5
	6- Being a human in the meeting with another human being	5, 7
	7- Acts of greeting each other or thanking each other	2, 4, 6
	8- Offering a human warmth	5, 7
	9- Increasing motivation and well-being—central for a “good day”	5, 9
	10- Creating a generous culture with non-existing hierarchy	4, 7, 9
	11- Feelings of trust	5
	12- Everyone's equal value	4, 9
	13- Colleagues contribute to create peace of mind	4, 7
	14- Inner harmony	7
	15- Feeling safe, and satisfied	10
	16- Proud of their role or position	4, 10
	17- Valuing oneself	7
Sharing togetherness in a greater whole, offering unconditional help, and devoting themselves to taking care of each other
	18- Accountability or relying on one another	1
	19- Responsible for one's colleagues	8
	20- To be close to rely on one another	1, 6, 7
	21- Righteous person, loyal	7
	22- Having genuine keenness to help others	7, 10
	23- To share knowledge and experience	2
	24- The virtue of being and feeling responsible for one another	1
	25- To give and take help	4
	26- Working side by side	4
	27- A feeling of togetherness	5, 8, 10
	28- Belonging	2, 5
	29- Being independent but still part of a whole	2, 7
	30- Not being alone	5, 10
	31- “Feeling at home”	4, 9, 10
	32- Involving one another	2, 3, 9
	33- Togetherness in a team	4, 5, 7
	34- Listening to each other—honest dialogs	3
	35- To give everyone a voice	2, 3, 4, 9
	36- Fairness towards each other's tasks	1
	37- Using a respectful language	4, 6

#### Creating a Mutual Bond on a Personal Level and a Permissive Atmosphere Through the Human Warmth

A health promoting relation between colleagues was created through an interpersonal bond between two people, a link between colleagues, through making personal connections, and through a mental understanding of each other (Aston et al., 2005; Schön Persson et al., 2018). This makes it possible to open-up to one's colleagues; to have a friend at work with whom one could share work-related thoughts (Arakelian et al., 2019), or share social and private issues if one desired (Schön Persson et al., 2018). In this kind of honest and friendly atmosphere, one dared to show one's inner feelings without judging or without being judged; it simply offered a human warmth (Schön Persson et al., 2018). This kind of friendship on a personal level contributed to increased motivation, the accomplishment of patient care, and well-being, and was central to a “good day” (Aston et al., 2005; Schön Persson et al., 2018). Humanity in the interpersonal relation referred to the quality of being a human in the meeting with another human being, by treating them well (Sastrawan et al., 2021; Schön Persson et al., 2018).

Furthermore, relationships were built between two people through socialization (Arakelian et al., 2019) or acquaintance (Trajano et al., 2017) through simple acts of greeting each other or thanking each other, small friendly talks (Arakelian et al., 2019; Hanssen et al., 2020; Tørring et al., 2019) during informal coffee breaks (Sastrawan et al., 2021; Schön Persson et al., 2018; Trajano et al., 2017), and helping to create a permissive atmosphere (Hanssen et al., 2020; Trajano et al., 2017), which led to good spirits between colleagues and humanization of the workplace (Arakelian et al., 2019; Hanssen et al., 2020). Those who created a good atmosphere among the colleagues were highly valued by all, and they were seen as role models (Hanssen et al., 2020; Sastrawan et al., 2021). It helped colleagues to work with a “twinkle in their eyes,” spreading joy and satisfaction in their work (Nunstedt et al., 2020). Fellowship and safety built an interpersonal culture through familiarity and knowing one another on a personal level (Arakelian et al., 2019; Tørring et al., 2019; Trajano et al., 2017).

Creating a generous culture with a non-existing hierarchy (Arakelian et al., 2019; Sastrawan et al., 2021), where barriers are broken down (Aston et al., 2005), allowed for a feeling of trust to be developed between the colleagues (Schön Persson et al., 2018), where everyone's equal value (Arakelian et al., 2019; Aston et al., 2005), regardless of their position (Arakelian et al., 2019; Trajano et al., 2017), was pointed out. Thus, colleagues contributed to creating peace of mind (Arakelian et al., 2019; Sastrawan et al., 2021), reaching inner harmony (Sastrawan et al., 2021), feeling safe, and satisfied with and proud of (Nunstedt et al., 2020) their role or position (Arakelian et al., 2019). This was based on valuing oneself and one's profession (Sastrawan et al., 2021), and a feeling of stability within oneself and the organization (Arakelian et al., 2019).

#### Sharing Togetherness in a Greater Whole, Offering Unconditional Help, and Devoting Themselves to Taking Care of Each Other

Accountability or relying on one another was described as a virtue to cherish (Bakhtiari et al., 2020). When entering a room with one's colleagues, one was responsible for not only the patient but also one's colleagues. This meant being close enough to each other to be able to rely on one another (Hanssen et al., 2020; Sedig & Sibbald, 2021). Ethical behavior was about the virtue of being and feeling responsible for one another (Bakhtiari et al., 2020; Sastrawan et al., 2021); to be a righteous person, loyal, and compliant (Sastrawan et al., 2021); generous, humble, dedicated, determined, having self-resilience (Bakhtiari et al., 2020); and to dedicate oneself to transferring moral and social values, empathy, and ethical values to each other (Sastrawan et al., 2021).

Creating interpersonal relationships was also a result of working side by side with one's colleagues for a long time at the same workplace (Arakelian et al., 2019), through a feeling of togetherness or a sense of being a part of a bigger context (Nunstedt et al., 2020; Sastrawan et al., 2021) or belonging to a team/a group (Schön Persson et al., 2018; Tørring et al., 2019). This was described as each person being independent but still part of a whole, which was also expressed as not being alone or “feeling at home” (Arakelian et al., 2019; Aston et al., 2005; Nunstedt et al., 2020).

Developing interpersonal relations meant having a genuine keenness to help others, that is, giving unconditional help (Nunstedt et al., 2020; Sastrawan et al., 2021), and to share knowledge and experience (Tørring et al., 2019), which were essential for personal commitment (Bakhtiari et al., 2020). To teach one another and having the more experienced ones help the newcomers helped to establish good dynamics in the organization; it became a part of the culture in the organization (Sastrawan et al., 2021). Instructing or teaching (Trajano et al., 2017) meant to give and receive help (Arakelian et al., 2019; Schön Persson et al., 2018; Tørring et al., 2019), to transfer ethical knowledge not only to one's colleagues (Bakhtiari et al., 2020) but also to interdisciplinary learning (Aston et al., 2005; Tørring et al., 2019). Furthermore, it was about providing comments on each other's performance (Trajano et al., 2017), giving a possibility to develop oneself and others (Arakelian et al., 2019). In technically and socially complex organizations, everyone is dependent on each other's knowledge, and they strive to reach personal and organizational excellence (Hanssen et al., 2020).

Key terms of interpersonal relations described in this theme include: respect for each member's tasks and responsibilities (Hanssen et al., 2020; Sastrawan et al., 2021) and roles (Bakhtiari et al., 2020; Trajano et al., 2017) in the team; sharing the same views and goals (Arakelian et al., 2019; Aston et al., 2005; Hanssen et al., 2020; Schön Persson et al., 2018), and involving one another (Aston et al., 2005; Tørring et al., 2019; Trajano et al., 2017) in solving problems; gathering thoughts to be on the same page (Aston et al., 2005); or making decisions together (Nunstedt et al., 2020; Trajano et al., 2017). Togetherness in a team also meant being genuinely engaged in working together and enjoying it (Sedig & Sibbald, 2021; Seligman, 2011); listening to each other, based on honest dialogs (Trajano et al., 2017); and giving everyone a voice and an opportunity to have the confidence to express their opinions (Arakelian et al., 2019; Aston et al., 2005; Tørring et al., 2019; Trajano et al., 2017).

Silent interpersonal dynamics occurred when people felt safe and secure in each other's company (Tørring et al., 2019) in the team. Respect for human dignity was equated with respect for personal identity and fairness toward each other's tasks, and effective interactions, meaning that respect for human dignity increased effective interactions between the team members (Bakhtiari et al., 2020). When one showed respect for one's own dignity (culture, autonomy, and physical space), the person was more likely to show respect toward others. Respect could be shown verbally, that is, using a respectful language and by having confidence in one another (Hanssen et al., 2020), and nonverbally, that is, shown through action (Tørring et al., 2019).

### Synthesized Abstraction

Thirty-seven subthemes emerged, which flowed into two main themes. The main themes were abstracted into metaphorical mental and physical implications of the home/homeness as part of the colleagues sharing the same reality. From these findings, the metaphor: “Healthy relations between colleagues at work constitute the community as a common world, encompassing feelings of being at home, implying acting in an expression of the ethos and dignity, a culture created that makes the ideal form of healthy relations visible” emerged.

## Discussion

In this meta-synthesis, the findings showed that ethical behavior included the virtue of being and feeling responsible for one another, something that Wärnå et al. (2007) highlighted as being important for employees’ health. The findings also indicated that honesty was seen as important, related to sincerity, truthfulness, and righteousness with respect to the way one presents oneself to others (Wärnå et al., 2007). Another important finding was the presence of a friendly atmosphere. In working life, it is recognized by a shared friendship and the joy that comes as a result (Wärnå et al., 2007). Hilli (2007) points out that a culture characterized by tact, and humility is characterized by a friendship, which can support and offer concrete help when needed. Hilli (2007) emphasizes that friendship at work is based on what one has in common and in shared values. According to Wärnå et al. (2007), generosity enriches health by its content of sharing, and a will to do good. In conclusion, Wärnå et al. (2007) manifested the spirit of communion in work including the meaning of pride, honesty, and generosity, and respecting oneself and others.

The feeling of having something in common in a permissive atmosphere occurred when they used an approach of listening to each other in an honest dialogue. Damsgaard (2021) notes that developing healthy relations with empathy helps, including the ability to understand each other. Moreover, Damsgaard (2021) maintains that empathy and compassion grow with the ability to imagine other people's lives and fates and make colleagues at work capable of recognizing their own vulnerability. Another significant finding was feelings of trust and how trust was developed between colleagues. Mayeroff (2011) and Schön Persson et al. (2018) hold that if trust is to be created, it requires mutual trust, a must according to Mayeroff (2011). What is significant is that you must trust yourself to grow to be able to trust another to grow in his or her own time and way (Mayeroff, 2011).

In this meta-synthesis, the phenomenon of feeling at home was prominent. In her study on the metaphor of the home, Hilli (2007) and Vilhauer (2010) clarify a prominent aspect of healthy relations between colleagues at work as the feeling of being at home. A person who acts in freedom and inner harmony reflects an expression of being at home, which in that case can be interpreted as having consequences for the person's experience of health, an atmosphere, characterized by the freedom to be oneself (Hilli, 2007). According to Hilli (2007), it is the atmosphere of the home that determines how one lives there. To live means to be connected where you can flourish; a home that breathes peace, familiarity, and well-being. Seligman (2011) holds that flourishing is one of the five pillars of well-being. Flourishing in the workplace means individuals feeling good, being content, performing well, and finding a meaning at work.

### Strengths and Limitations

To discover meanings, dimensions, and connections that may remain concealed in purely empirical material, a synthesis with a hermeneutic approach was deemed appropriate (Bondas & Hall, 2007; Finfgeld, 2003; Sherwood, 1999). The fact that all participants’ experiences were considered assisted in broadening the horizon of studies that described healthy relations, creating health and well-being between colleagues at work. The synthesis demonstrates credibility, as the original data are credible because it represents true descriptions of human experiences (Finfgeld, 2003). Reliability and transparency were enhanced by describing the various steps in the synthesis as thoroughly as possible (Bondas & Hall, 2007; Finfgeld, 2003).

### Implications for Practice

Healthy relations are created when there is honesty, sincerity, truthfulness, righteousness, and respect between colleagues. Moreover, humanity, empathy, and generosity set the ground for a friendly and permissive atmosphere where support and help are offered between colleagues, and a friendship is created through honest dialogue and listening to each other.

## Conclusion

When the relations between colleagues are strong, it increases the meaning of their work, leading to an increased sense of well-being. Nurses find their “meaning” when they work in a permissive environment, and when they are allowed to be themselves around colleagues in communion. Such meaning can also be found through their engagement with one another, by sharing knowledge and offering each other unconditional help. Thus, colleagues contribute to each other's ability to flourish in their workplace. Good interpersonal relationships lead to a positive environment, a place people call their home, where there is trust and friendship. To create such a place, worthy of being called “home,” nurse colleagues must be provided time for co-reflections at their workplace, to create a friendly and welcoming atmosphere for all employees to coexist.
